# Molecular Dynamics Simulations of Hsp90 with an Eye to Inhibitor Design

**DOI:** 10.3390/ph5090944

**Published:** 2012-09-10

**Authors:** Elisabetta Moroni, Giulia Morra, Giorgio Colombo

**Affiliations:** Institute of Molecular Recognition Chemistry, CNR, via Mario Bianco 9, 20131 Milano, Italy; Email: elisabetta.moroni@icrm.cnr.it (E.M.); giulia.morra@icrm.cnr.it (G.M.)

**Keywords:** drug design, dynamic drug discovery, Hsp90, inhibitors, allostery, binding, molecular dynamics, simulations

## Abstract

Proteins carry out their functions through interactions with different partners. Dynamic conformational switching among different structural sub-states favors the adaptation to the shapes of the different partners. Such conformational changes can be determined by diverse biochemical factors, such as ligand-binding. Atomic level investigations of the mechanisms that underlie functional dynamics may provide new opportunities for the discovery of leads that target disease-related proteins. In this review, we report our views and approaches on the development of novel and accurate physical-chemistry-based models for the characterization of the salient aspects of the ligand-regulated dynamics of Hsp90, and on the exploitation of such new knowledge for the rational discovery of inhibitors of the chaperone.

## 1. Introduction

Increased knowledge about protein structures and interactions combined with an understanding of how specific regulatory networks change in pathological *versus* normal conditions can provide novel opportunities for drug discovery and design. Advances in both biochemical and computational techniques have provided remarkable insights into the description of such cellular networks. In particular it is now well established that most of the times proteins can function through direct associations with other proteins, while in more sporadic cases they can carry out their function as isolated entities. This information on protein linkages allows us to describe signalling pathways in the cell as modular networks. This representation, combined with the use of known three-dimensional structures to model the physical interactions among the nodes, is starting to enable researchers to build structure-based networks of the relevant protein complexes in normal and altered cells [1].

Exploiting such structure-enriched connectivity maps can clearly suggest relevant nodal proteins at the crossroads of different pathways as new drug-targets improving on the low yield, elevated costs, and high risk of failures of traditional drug screening aimed at inhibiting only one protein associated with one pathway. The networks can also help identify alternative drug targets that can be used to effectively disrupt a specific cellular pathway of virus or bacterial pathogens once it has become resistant to current drugs. Multiple regulatory cascades can indeed be converged onto a single nodal-target whose inhibition can affect multiple signaling pathways resulting in cell death, since proteins with high connectivity are more likely essential for cell survival. In contrast, the inhibition of the proteins responsible for each of the separate signaling processes would require the administration of multiple drugs [2].

The 90 kDa heat shock protein (Hsp90) is a paradigmatic example of a protein that mediates multiple networks responsible for signaling cascades that are often associated with the development, maintenance and invasiveness of cancer cells. Hsp90 is a promiscuous molecular chaperone that directly oversees the correct conformational maturation, activation and maintenance of a plethora of proteins associated with all six hallmarks of cancer: angiogenesis, immortalization, metastasis, impaired apoptosis, insensitivity to antigrowth signals, autocrine growth [3,4,5]. Consequently, the study of Hsp90 function and inhibition has been actively pursued over recent years in search for new cancer chemotherapeutics.

The promise of Hsp90 inhibition is highlighted by the fact that as much as 13 molecules are involved in Phase I/II clinical trials for the treatment of various cancers [6]. In this context, it was shown that Hsp90 in cancer cells (BT474, N87, SKOV3, and SkBr3; average IC50 ~5 nM) has a higher affinity for ligands than Hsp90 in normal cells (normal dermal fibroblasts, human renal epithelial cells, HMVEC, HUVEC, Hs578Bst, and PBMC; average IC50 ~943 nM). The enhanced affinity of tumorigenic Hsp90 for ligands results from the high population of Hsp90-client complexes found in cancer cells, which are strictly dependent on the Hsp90 machinery for continual growth in hostile microenvironments [7]. In normal cells, Hsp90 can be found in its unbound, inactivated state, with lower affinity for ligands [8]. These data support the hypothesis that Hsp90 inhibitors can be made selective for cancer cells.

In line with what was discussed above, disruption of the Hsp90 machinery simultaneously inhibits multiple enzymes essential for cancer proliferation. As a consequence, it is no surprise that this molecular chaperone has become a prime target for drug development [6].

**Figure 1:**
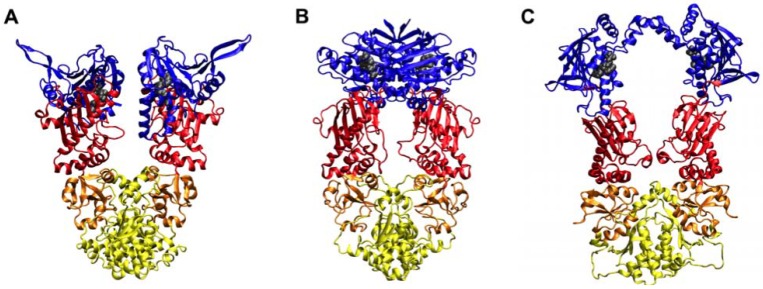
(**a**) 3D structure of Grp94; (**b**) 3D structure of Hsp90; (**c**) 3D structure of HtpG.

The chaperone function of Hsp90 depends on ATP binding and hydrolysis. From the structural point of view, Hsp90 is a homodimer in which each protomer is characterized by a modular architecture with three well-defined domains: an *N*-terminal regulatory Domain (NTD), responsible for ATP binding, a Middle Domain (M-domain), which completes the ATPase site necessary for ATP hydrolysis and also binds client proteins, and a *C*-terminal Domain (CTD) that is required for dimerization and allosteric regulation of the *N*-terminal ATP-binding site (Figure 1). The first X-ray crystal structures of full-length Hsp90 from yeast bound to the ATP mimic, AMP-PNP, revealed a “closed” conformation in which the two NTDs dimerize in a compact structure. The same global topology is shared by the endoplasmatic reticulum (ER) paralog, Grp94 and by the bacterial homolog HtpG, although the relative orientations of the N- and M-domains vary significantly depending on the organisms [9,10,11,12].

Solution state studies using small angle X-ray scattering (SAXS) evidenced the highly dynamic and stochastic nature of the chaperones, in which the equilibrium between different conformational states can be readily shifted to adopt conformations that are suitable for co-chaperone recognition and modulation of ATPase activity. Furthermore, it appears that nucleotide binding may shift this equilibrium towards compact conformations. Importantly, equilibrium and kinetic measurements showed that conformational transitions during the chaperone cycle can be structurally similar among different Hsp90 proteins, while the energetic balance between individual steps may be species-dependent [12,13,14,15,16,17,18]. These observations suggested a role for the nucleotide in modulating the conformational preferences of the protein, related to specific functions in the chaperone cycle.

Overall the functional mechanism of Hsp90 can be viewed as a conformational cycle, coupled to ATP binding and hydrolysis, that involves the opening and closing of a dimeric molecular clamp formed by the transient association of the NTDs in the homodimer owing to the constitutive association of highly conserved motifs in its CTDs. ATP binds to the NTDs of Hsp90, stabilizing their transient dimerization [15]. Although the exact mechanism of coupling between ATP-binding/hydrolysis and client protein folding remains unclear, structural and biochemical data support a picture in which nucleotide binding at the NTD propagates a conformational signal to the CTD and the chaperone undergoes structural rearrangements that bring the two NTDs into close association in the ATP-bound state, but not in the ADP-bound or apo states. The CTDs act as hinges for the “molecular clamp” mechanism. Based on these mechanistic considerations, several attempts have been initially performed to inhibit Hsp90 by blocking ATP binding to the nucleotide binding pocket in the *N*-terminal domain.

All drugs in clinical trials are actually *N*-terminal inhibitors: they include derivatives of the natural products geldanamycin (GA) [19,20], radicicol [21]; of a new class of synthetic compounds mimicking ATP [22]; and of the 3,4-diarylpyrazole, CCT018159 [23]. Unfortunately, clinical trials showed that the drug concentration required to induce client degradation is the same needed to induce the heat shock response, which results in the overexpression of both Hsp70 and Hsp90. Since both Hsps are pro-survival [24,25,26], the induction of Hsp70 and Hsp90 determines an unfavorable condition in which the patients require higher doses at an increased frequency that eventually translates into tolerance and toxicity problems.

Recent studies demonstrated that the Hsp90 CTD is essential for dimerization of the chaperone and contains an additional binding site [27]. Inter-domain communications between the Hsp90 NTD and CTD may trigger a conformational change in the CTDs and their interfaces, unveiling an otherwise hidden *C-*terminal binding site [12]. Indeed, initial studies directed toward inhibition of the Hsp90 machinery by novobiocin and related leads, showed selective binding to the CTD and not the NTD, leading to the destabilization of Hsp90-cochaperone complexes with concomitant degradation of several clients [28]. Novobiocin was also found to destabilize the multiprotein complex necessary for client folding, resulting in ubiquitinylation of the client protein, proteasome mediated hydrolysis [27], and in many cases, induction of apoptosis. Cox *et al.* demonstrated that *N*-terminal inhibitors were unable to overcome the effects of overexpressed p23, because they competed for the same region. In contrast, they found that inhibition of the *C-*terminal binding domain with novobiocin was independent of p23 concentration. Consequently, *N-*terminal inhibitors would be less effective in tumors that overexpress p23, which may also provide an origin for resistance [29]. Therefore, more potent inhibitors of the *C-*terminal binding site are likely to produce compounds with activity against a wide range of cancers, including those that overexpress p23. Furthermore, compounds that inhibit Hsp90 function without inducing Hsp expression would alleviate the dosing and scheduling difficulties arising in clinical trials with *N-*terminal inhibitors. Several derivatives that target the *C-*terminal domain, initially based on the novobiocine scaffold, have been synthesized by the Blagg group and helped define important structure activity relationships and showing promising anticancer activities [30,31,32].

Altogether, the structural, recognition and inhibition information available for Hsp90 point to a key role of the ligand-regulated flexibility of the chaperone in determining its functional properties. In this context, it is clear that shedding light on all of these aspects at atomic resolution is critical for both fundamental and practical reasons. From the fundamental point of view, understanding why the binding of a specific nucleotide or drug molecule translates into specific functionally oriented protein motions can help in getting insights into the molecular basis ligand-regulated protein dynamics and conformational selection phenomena as well as deepening our understanding of the relationships between protein structure, dynamics and functions. From the practical point of view, these aspects can be extremely useful in the development of new rules for drug selection, discovery and design with possible important implications for the development of novel pharmaceuticals.

In this paper, we will review and discuss our approaches aimed at integrating the atomic-resolution investigation of the determinants of Hsp90 conformational dynamics with novel approaches for drug discovery and design. Studies based on atomistic simulations have been successfully used for understanding the regulation of conformational dynamics of this [33] and other systems [34,35].

Our studies are carried out using molecular dynamics (MD) simulations of isolated Hsp90 domains or of the full-length Hsp90 dimer, combined with the analysis of ligand effects on the conformational plasticity, of the essential conformational modes and of residue-pair coordination properties across the structures. Such analyses are integrated to generate new strategies for the identification of allosteric sites of protein functional regulation, which can then be used as druggable targets in the discovery of new allosteric leads. In general, molecular simulations have been successfully used to investigate enzyme reactivity and selectivity [36,37,38,39,40,41], the factors governing structural stability and functional dynamics [42,43], in drug design projects [44,45,46,47] and to investigate folding and misfolding [48,49].

This review is organized as follows: a first section is dedicated to the analysis of structure-based computational studies of the isolated *N-*terminal domain of Hsp90 for the design and development of new Hsp90 inhibitors and for the generation of models that help explain the effects of different ligands on the conformational dynamics of the domain. Next, we will discuss all-atom studies of the full-length protein aimed at understanding how the signal encoded by the nucleotide binding at the *N-*terminal domain is propagated to the *C-*terminal domain, regulating the molecular clamp motion. This analysis is then extended in a comparative manner to several, structurally different representative members of the Hsp90 family to highlight common aspects of functionally oriented, large-scale conformational changes. These results are used to develop a unified, atomic resolution model of ligand-regulation of dynamics and allosteric signal propagation in Hsp90s.

This analysis and the development of new models to describe the global and allosteric dynamics of Hsp90 set the basis for the development of new methods for drug selection that take conformational dynamics explicitly into account. We will discuss dynamics-based approaches in the identification of new hits that can target the *N-*terminal nucleotide-binding site or novel allosteric binding sites. We conclude by discussing new perspectives and possible future directions in the use of molecular simulations for the design of improved Hsp90 inhibitors.

## 2. Studies of the Isolated Hsp90 *N-*terminal for Drug Development

### 2.1. MD Simulations in the Study of the Recognition between Hsp90-NTD and Ligands

Our studies concerning the development of Hsp90 targeted molecules using all-atom Molecular Dynamics (MD) simulations started with the design and engineering of shepherdin, a cell-permeable peptidomimetic, modeled on the binding interface between the molecular chaperone Hsp90 and the antiapoptotic and mitotic regulator, survivin [50].

Shepherdin was initially developed using synthetic peptidyl mimicry, which identified the K79–K90 sequence of survivin as the sequence needed to block the interaction between survivin and Hsp90, *in vitro* [51]. Surface plasmon resonance analysis showed that the peptide could bind Hsp90 with high affinity (K_D_ ± SEM = 8.38 × 10^−8^ ± 3.5 × 10^−9^ M) at increasing ligand concentrations (0.1–10 μM). Variable sequences were subsequently tested and permitted to identify sequence K79–L87 as the minimal motif sufficient to bind Hsp90. A retro-inverso peptidomimetic analog designated shepherdin-RV (inverse sequence L79–K87, all D amino acids) was also designed, with the aim of increasing protease resistance.

**Figure 2:**
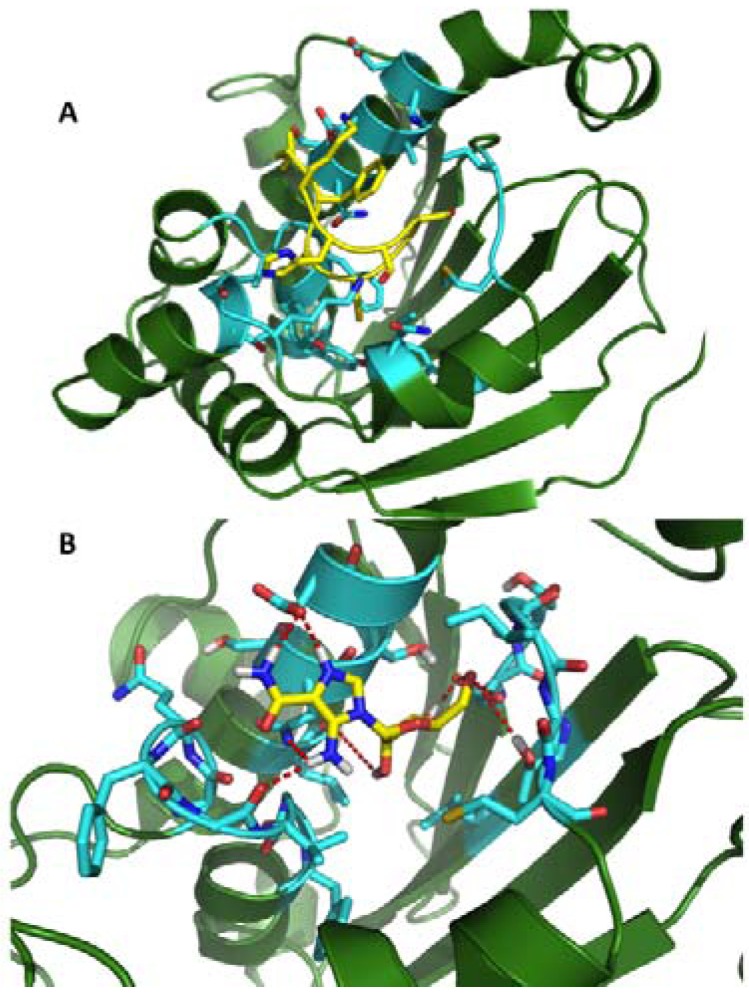
(**a**) Structural representation of the complex between shepherdin (yellow) and the *N*-terminal domain of Hsp90. The Hsp90 residues involved in the most stable interactions are depicted in light blue; (**b**) Structural representation of the complex between AICAR (yellow) and the *N*-terminal domain of Hsp90.

Extended all-atom, explicit solvent MD simulations were first used to characterize the conformational properties of shepherdin and shepherdin-RV in isolation. Both peptides were found to populate a dominant configuration characterized by a turn involving S82–G83 in shepherdin and G83–S84 in shepherdin-RV, and overall β-hairpin geometry (Figure 2). The representative β-hairpin conformations of shepherdins were subjected to multiple blind docking experiments on the amino-terminal domain of Hsp90 using the AutoDock program package [52]. In all cases, the peptides were predicted to dock into the ATP binding site of Hsp90 (Figure 2). Shepherdin and shepherdin-RV make 13 and 18 predicted hydrogen bonds with the ATP pocket of Hsp90, respectively, involving the side chains of H80, S81, S82, the carbonyl group of G83, and the side chains of K87 and C82 (shepherdin-RV). Except for D93, the complementary residues of Hsp90 predicted to make contact with shepherdin and/or shepherdin-RV largely overlap with amino acids implicated in GA binding [53].

The results of the structural predictions were validated experimentally through targeted mutations in the ATP pocket of Hsp90, which were predicted to have an effect on shepherdin binding. Individual substitution of N51, S52, D102, or S113 in the N domain of Hsp90 reduced binding to shepherdin by 20%–60%, whereas mutagenesis of “GA-specific” D93 had no effect, and a scrambled peptide did not bind wild-type or mutant Hsp90 [50].

In subsequent *in vitro* and *in vivo* tests, shepherdin was shown to destabilize Hsp90 client proteins, and induce massive death of tumor cells by apoptotic and nonapoptotic mechanisms. Importantly, shepherdin did not influence the viability of normal cells. *In vivo*, shepherdin was well tolerated, and could inhibit human tumor growth in mice without toxicity. Shepherdin proved to be an extremely interesting candidate for the development of potent and selective anticancer agents in humans.

### 2.2. Dynamic Pharmacophores

The structure of the Hsp90-shepherdin complex was next used as starting point for the development of a novel and general approach based on the combination of docking and all-atom MD simulations aimed at the identification of low molecular weight compounds that may act as structurally novel antagonists of the chaperone. This dynamic information permitted to consider the mechanisms by which the ligand and the binding site adapt to each other and, as a consequence, it was possible to build a pharmacophore model for virtual screening which takes into account accessible conformations of both the receptor and the known active ligand in the bound state [54].

In this specific case, the structure of the Hsp90/shepherdin complex was subjected to two long, 54 and 73 ns, all-atom MD simulations. During the simulation shepherdin partially reoriented to increase the total number of stabilizing contacts with the ATP binding pocket. Analysis of the statistical and time-dependent distribution of the interactions between functional groups of the ligand and of the chaperone determined through MD was next used to develop pharmacophore models, which keep explicitly into account the motional and flexibility properties of both the ligand and the receptor. In this analysis, as well as in subsequent applications of the approach, hydrogen-bonding, hydrophobic/aromatic and charge-charge interactions were monitored and used to develop the pharmacophore model as they represent the most common types of intermolecular forces determining drug/protein molecular recognition. The conformation of shepherdin and the orientations of its side-chain functional groups in the most populated structural cluster from MD trajectories of the complex were used as structural template (Figure 2). The distributions of dihedral values and distances among critical functionalities obtained from the full MD trajectory analyses were used to define upper and lower boundaries for geometric constraints. This was considered equivalent to including variations and fluctuations among different functional groups in the pharmacophore design.

The resulting pharmacophore models were used to screen the NCI 3D database and returned about 20 hits with drug-like properties. Most of these molecules were characterized by the presence of purine like scaffold similar to the ones of already known Hsp90 inhibitors. Interestingly, one of the non-purine-based hits that was found to be effective in experiments, AICAR (5-aminoimidazole-4-carboxamide-1-*β*-D-ribofuranoside) was not previously known to interfere with Hsp90 chaperone functions and was characterized by a novel molecular structure among Hsp90 antagonists.

Successive experimental tests showed that AICAR could inhibit Hsp90 chaperone function with destabilization of multiple client proteins, including Neu, CDK6, Akt, survivin and telomerase. The molecular inhibition of Hsp90 chaperone functions translated into antiproliferative and proapoptotic activity in multiple tumor cell lines, while not affecting proliferation of normal human fibroblasts. AICAR thus emerged as a possible a viable lead for further development of anticancer drugs [54].

In this work, we showed that that the use of pharmacophore models derived from a statistical analysis of the dynamics of binding and the avoidance of constraints linked to the use of defined chemical scaffolds, can actually expand the chemical diversity of the predicted ligands. It is worth noting that dynamics-based pharmacophore models can be immediately used to screen libraries of drugs already approved for different uses in human pathologies, significantly shortening the distance separating hit discovery from clinical application and overcoming the time lag needed to carry out safety tests. These considerations could be particularly relevant in the development of strategies for drug repositioning and for the study of secondary and off-target effects.

Next, docking studies followed by all-atom MD simulations were used to investigate the structure of the complex between AICAR and Hsp90. The information obtained from the simulations was combined with NMR analyses of the complex and of the small molecule isolated in solution [55]. This endeavor was undertaken based on the consideration that long time scale MD simulations provide a general view of the different possible states accessible to the ligand in isolation or in the bound state. NMR analysis helps select and filter only those conformations that verify specific structural constraints measured in solution at equilibrium, recapitulating ensemble properties that are specific only to selected molecular configurations. The end result is the generation of experimentally validated conformational ensembles of the ligand in isolation and in the complex, which may be particularly useful in the presence of large receptor proteins, not yet amenable for full NMR analysis or in the presence of highly flexible ligands, for which X-ray may fail to provide an atomic structure.

The data showed that in the AICAR bound state one conformation of those present in solution is selected, where imidazolic H4 and H5 protons have a key role in defining a non polar region contacting Hsp90 surface. The dynamic equilibrium between two possible conformations of AICAR ribofuranoside moiety, namely the S-type and N-type puckered forms, was also shown to be functional to inhibitor binding. Indeed, NMR-based *J*-coupling measurements suggested that in the free state the most populated conformation appeared to be the S-type, whereas the conformation preferred by bound AICAR was the N-type. The modulation of conformational equilibrium upon binding could also be extrapolated from the MD trajectories of free and bound ligand, with the S-type population representing the preferred conformation for AICAR in solution, while the N-type appeared to be prevalently populated in the complex with Hsp90-NT. The combined NMR and MD data thus revealed a conformational selection model for AICAR binding to Hsp90 [55].

The combination of computational biology and NMR approaches for relatively fast and reliable determination of protein-ligand structural models, keeping into explicit account the roles of conformational flexibility as well as the possibility for a certain bimolecular complex to visit alternative configurations available on the energy landscape, provide important pieces of information in drug-design. In designing a molecule with specific properties, it is fundamental to be able to select the native-states representatives (most populated configurations corresponding to free energy minima on the energy landscape) from non-native ones and to detect possible differences in the structural dynamics of the ligand when in the free state and when complexed to the receptor. In the drug-design context, the combined MD-NMR approach singles out the relevant groups involved in interaction, with a clear indication of the importance of the different moieties.

These data can be used to validate and improve pharmacophore models for the rational identification/design of new and more active Hsp90 inhibitors as new anticancer therapeutic candidates.

## 3. Studying Ligand-Based Modulation of Hsp90-NTD Dynamics

The investigation of the complexes between shepherdin or AICAR and Hsp90-NTD provided insights into the fundamental importance of conformational dynamics in molecular recognition and molecular design. These data were complemented by simulations of the domain in complex with the natural ligands ATP and ADP as well as in the apo state. The results showed that the different binding partners could modulate the conformational dynamics of Hsp90-NTD linked to the functional activities of the molecular chaperone [56]. In particular, the active site lid was shown to act as a conformational switch, populating different conformations in the presence of different ligands. This in turn translated in the modulation of the global dynamics of the whole domain. In the context of the conformational selection model of binding, the model we proposed implies that the apo Hsp90-NTD populates a structural ensemble with a range of possible distinct lid conformations. Ligand binding modulates the equilibrium among conformational states to different extents that depend on the ligand identity, leading to a diversity of functionally relevant complexes. Specifically, ATP-binding leads to a highly constrained structure whose formation involves coupled conformational switches in the *N*-terminus and lid, which folds and closes onto the nucleotide binding pocket, leading to the “tense” native structure. In contrast, highly flexible and “relaxed” states are observed in the complexes with the inhibitors, in which the lid populates a number of different conformations.

Overall, the results of this study suggested that the activity of the Hsp90 can be modulated by the local conformational transitions and switching of the *N*-terminal “lid” as a function of the binding partner that reverberate in differences in the protein dynamics as a function of the binding partner. The emergence of ligand-based modulation of the Hsp90 N-domain conformational dynamics supports the mechanism of the active site region and of the active site lid as a nucleotide sensitive conformational switch of the molecular chaperone activity. Hence, through these mechanisms of dynamic regulation, the local effects determined in the Hsp90 *N*-terminal may be transmitted across the protein structure, allowing functional diversity of the protein structures [56].

## 4. MD-Simulations of the Full Length Hsp90: Identification of Possible Allosteric Pockets and Allosteric Drug Design

The observation that the structural plasticity of Hsp90 in complex with ATP, ADP and inhibitors can be exploited by the chaperone machinery to modulate enhanced structural rigidity during ATP binding and increased flexibility upon inhibitor binding [56], raises the question whether these effects are limited to the NTD or can diffuse across the full-length protein structure. In this line of thought, all-atom MD simulations were used to study the extent to which distinct subdomains of the *full-length* Hsp90 are mechanically coupled, and hence capable of propagating signals that, upon ligand-binding to a specific site, cause conformational responses in distal regions [57]. The examination of the conformational dynamics of the apo, ATP- and ADP-bound states of *full-length* Hsp90 based on the analysis of correlated motions revealed that positive long-range cross-correlations among pairs of residues could extend well beyond local elements and intra-domain regions in the ATP-state, indicating the presence of a diffuse interaction network spanning the whole structure [57]. The functional consequence is the stabilization of the closed “tense” state by favoring the Hsp90 dimer complex to move as a coherent rigid body. In the ADP-state the positive correlation was lost and the two protomers moved in an anti-correlated way. This indicated collective motions towards opposite directions, setting up the consequent opening of the clamp.

In this context, we developed a general theoretical model to identify the “hot spots” active in the allosteric communication between a certain binding site and distal regions of the protein implied in function. The cross-talk between the N- and C-domains was investigated by defining the communication propensity (CP) between any two residues as a function of fluctuation of their distance components. CP describes a communication time, therefore low CP values are related to efficiently communicating residues. Hot spots for allosteric transduction were identified by calculating for each residue the fraction of all other residues that have high communication efficiency with it at distances larger than increasing cutoffs [57]. This analysis illuminated different allosteric pathways that selectively depend upon the identity of the ligand in the *N*-terminal domain: specific clusters of residues participate in the signal transduction from the *N*-terminal binding site to the CTD. In the ATP-bound, active state, long-range communication from the binding site was directed to residues at the CTD interface.

It must be underlined that evidence for efficient molecular communication between NTD binding site residues and CTD regions in the activated state is of special importance given the growing interest in developing novel and specific inhibitors targeting allosteric Hsp90 regions.

The hot spot *C-*terminal region was *then* subjected to structural investigation and one pocket (labeled Pocket A) was consistently detected in all representative MD conformations, located at the dimer interface and found to be suitable to accommodate small compounds that interact directly with the hot spot allosteric residues. The information on signal transduction, on the conformational states spanned by hot spots in Pocket A, and the analysis of their chemical properties were used to develop pharmacophore models for virtual screening of drug databases [58].

**Figure 3:**
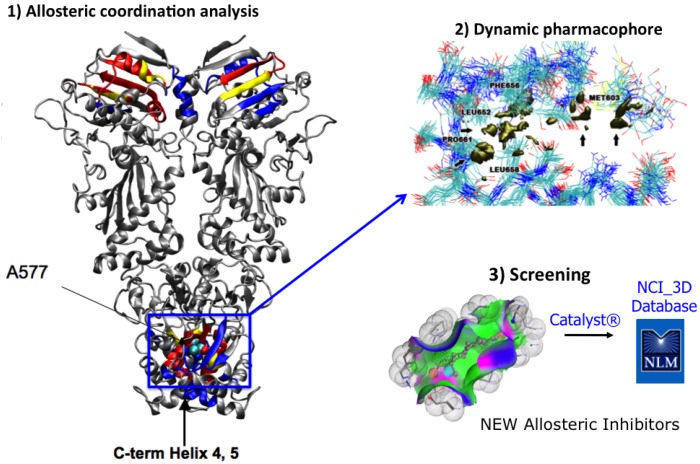
Scheme recapitulating the combination of allosteric site identification and pharmacophore development for virtual screening.

The pharmacophore recapitulated the complementary interactions necessary to guarantee productive binding with the putative allosteric site, and was used to screen the NCI repository. Out of 290,000 compounds, filtering with the pharmacophore returned 36 hits, corresponding to 0.01% of the database. These compounds showed experimental activities in line with the computational design. Namely, they showed affinity for the Hsp90 *C-*terminal domain, had effects on the viability of cancer cells while leaving normal cells unscathed, induced degradation of specific Hsp90 client proteins, and could disrupt Hsp90 association with co-chaperones [58] (Figure 3).

Importantly, the results of the experimental tests showed the feasibility of using computational methods to identify protein regions important for allosteric regulation, based on a rational model of long-range mechanical coupling among protein residues. Small molecules targeting the putative allosteric hotspots showed the ability to perturb the chaperone’s functional dynamics with the consequent inhibition of several important protein-protein interactions with clients and co-chaperones, demonstrating the ability to interrupt biological pathways fundamental to cancer cell proliferation.

The possibility to glimpse at the determinants of relevant dynamic properties underlying protein function at atomic levels of resolution, the success of dynamics-based pharmacophores in identifying new compounds with promising pharmacological properties and the ever-increasing computational power available through technological and algorithmic advances are starting to make the use of multiple receptor conformations in drug discovery an affordable complement (if not an alternative) to classical High Throughput Screening (HTS) efforts based on static structures.

These concepts were employed in the development of a novel rational strategy aimed at selecting new *N-*terminal targeted inhibitors of Hsp90, starting from the atomistic analysis of the conformational dynamics of the complex between the full-length protein and its natural ligand ATP. This complex represents the activated state of the chaperone. 

**Figure 4:**
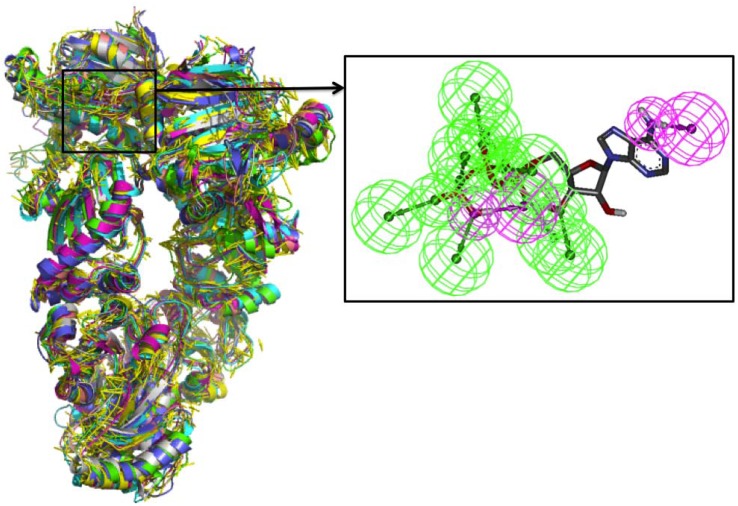
Superposition of the different substates accessible to Hsp90 in the presence of ATP and the corresponding derived pharmacophore.

The dynamics-based approach starts from the identification of the relevant representatives of distinct conformational substates visited during a long timescale molecular dynamics (MD) simulation of the complex between full length Hsp90 and ATP [59] (Figure 4).

This is obtained through the application of a clustering procedure that identifies the relevant representatives of distinct conformational substates from an MD simulation and is based on the *K*-medoids clustering scheme. This technique considers as input only the pairwise RMSD distances between all the pairs of recorded structures and provides a pre-assigned number *K* of non-empty and homogeneous clusters, each of them characterized by a representative conformation. The method identifies the clusters iteratively minimizing a “dissimilarity score” obtained by summing the RMSD of each structure from its cluster representative. The standard *K*-medoids technique relies only on geometrical considerations. Each cluster was forced to contain structures that span a continuous time-interval. This aspect was fundamental to ensure partitioning of the MD trajectories into a series of progressively visited substates. The aim of this step was to select a minimal number of key configurations that capture most of the structural fluctuations encountered in the multi-nanosecond trajectory, recapitulating the flexibility of the complex and providing a compact representation of how internal dynamics reverberates on the types and persistence of intermolecular interactions between Hsp90 and ATP in the *N-*terminal domain. The bound nucleotide was used as a template for pharmacophore design. The analysis of the interactions between the nucleotide and the binding site and their fluctuations within different substates was used to build a pharmacophore model in which the relative importance of each pharmacophoric feature is weighted according to the conservation of the interaction it mimics in the different substates. The resulting pharmacophore could then take into account in a simple and compact manner the effects of protein mobility on ligand binding and the reciprocal effect of the nucleotide on the internal dynamics of the protein. Subsequent screening of a small molecule database identified novel antagonists targeting the *N-*terminal domain promising anticancer activity. The compounds indeed showed activity against human breast carcinoma cell lines, decreased the expression levels of Hsp90 client proteins AKT, CDK4, CDK6, and surviving and showed the induction of apoptosis in treated cells [59].

These results once more provided support to the idea of including flexibility in drug selection strategies and gave a clear illustration of how the knowledge of the internal dynamics of a system can be exploited to expand the molecular diversity space of possible active ligands.

### Comparative Dynamics

The study of the ligand-modulated dynamics of Hsp90 can be further expanded by approaching the comparative dynamical analysis of multiple Hsp90 homologue proteins [60].

The search for novel allosteric sites in Hsp90 can take advantage from the parallel study of the conformational dynamics of different members of the protein family, for which both significant common traits as well as species-dependent features are known. Those aspects were addressed by analyzing and comparing atomistic simulations of Hsp90 family representatives for which crystal structures of the full length protein are available: mammalian Grp94, yeast Hsp90 and *E. coli* HtpG. These chaperones were studied in complex with the natural ligands ATP, ADP and in the apo state. Key, common aspects of their functional dynamics were elucidated with a multi-scale comparison of their internal dynamics. In particular, starting from the atomic resolution investigation of internal fluctuations and geometric strain patterns, a novel analysis of domain dynamics was developed based on a decomposition into rigid domains. Namely, distance fluctuation maps, reporting on the internal rigidity of protein subdomains in each system, suggested that the chaperones’ internal dynamics primarily results from the relative motion of a limited number of quasi-rigid domains, involving a set of residues that can be put in one-to-one correspondence among the proteins. The simulated MD trajectories were filtered by retaining only the Ca atoms of the subset of corresponding amino acids and combined into a “meta-trajectory” spanning a total of 1,800 ns (900 ns per protomer). The fluctuations of pairwise Ca distances in the meta-trajectory were used to optimally divide the corresponding amino acids into quasi-rigid domains. It was found that as few as three-rigid domains suffice to describe well the conformational space spanned by the meta-trajectory. The main corresponding large-scale movements across the three chaperones could be represented by restraining the motion of the two terminal quasi-rigid domains to pure rotations around an axis that is fixed with respect to the central domain.

Two common primary hinges for such movements are hence identified. The first hinge site, whose functional role has been demonstrated by several experimental approaches, is located at the boundary between the *N-*terminal and Middle-domains. In this respect, the *N-*terminal quasi-rigid domain matches one of the standard structural domains, namely the *N-*terminal one. In contrast, the *C-*terminal rigid domain includes part of the structural Middle domain: specifically, the second hinge site is located at the end of the H4-H6 three-helix bundle at the interface between the M-large and M-small structural subdomains.

The results show that, in spite of the diversities in the initial structural arrangements of the molecules, a common mechanism presides their ligand-dependent large-scale motion. In fact, the hinge located at the interface between the M-large and the M-small domains is highlighted in all proteins as a mechanical hotspot that is capable of modulating the motion of the *C-*terminal domain in a ligand-dependent manner. It is observed that the hydrophobic core ensuring stability to the H4-H6 helix bundle increases its motion and unfolds/unpacks going from the ATP- to the ADP-state. It is worth stressing that such key mechanical role is not evident from the standard structural partitions of the protomeric domains. This site could therefore represent a promising novel druggable allosteric site common to all chaperones [60].

## 5. Hsp90 Protein-Protein Interaction Networks and Drug Design

To this point, we have presented and discussed a set of results in which we have considered Hsp90 as a single molecule. It has been estimated that the chaperone may assist the folding of up to 10% of all cytosolic proteins at some stage of their life cycle [61]. Moreover, Hsp90 functions are regulated through interactions with a plethora of regulators and co-chaperones.

A large number of experimental efforts based on biochemical and genetic approaches have been used to build maps of the Hsp90 interactome [62]. The Picard group has recently proposed a novel approach to elucidate Hsp90 interaction networks in a comprehensive way [63]. The authors developed a workflow for parallel mining of all major PPI databases, containing data from several model organisms, and to integrate data from the literature. The workflow was validated by experimentally proving the prediction that the Hsp90 co-chaperone Aha1 is involved in nucleocytoplasmic transport. Several intersections with other PPI networks could be characterized.

In this context, it is worth noting that a previous report aimed at characterizing Mitogen-activated protein kinase (MAPK) pathways found links with Hsp90 [64].

In a different, yet related context, Bergman and Siegal [65] used numerical simulations of gene networks combined with genome-scale expression data from yeast single-gene deletion strains, to investigate the role of Hsp90 as evolutionary capacitor, alone or in combination with other proteins. The results suggested the existance of a large class of evolutionary capacitors whose effects on phenotypic variation complement the systemic effects of Hsp90.

Overall, these data suggest new opportunities for the development of inhibitors aimed at interfering with specific protein-protein interactions. However, a number of hurdles must be overcome before obtaining effective leads for drug development. First, a very limited number of complexes between Hsp90 and partner proteins has been solved at high resolution. In many cases, only subdomains of the chaperone are present in the crystal. The recent NMR characterization of the interaction between Hsp90 and p53 by NMR spectroscopy by Kessler and coworkers [66] is an important step forward in this case. The second problem is represented by the fact that protein-protein interaction surfaces are rather large and flexible. Finding a small molecule that may efficiently target such surfaces may in general be more difficult than classical drug design in which a small molecule has to target a well defined pocket with specific and rigid stereoelectronic properties. A possible lead, however, does not have to cover the entire binding area. Binding pockets have hot spots that largely determine binding. The problem is how to find them.

In such cases, computational approaches aimed at predicting binding hotspots [67] on protein surfaces may certainly be of great utility. The identification of such hotspots on Hsp90 can be related to its interactions with other proteins in a certain pathway. The inhibition of protein-protein interactions can thus be used to perturb (part of) a specific network and to determine the functional consequences of the perturbation.

## 6. Conclusions

Proteins perform their functions and participate in cellular networks through interactions with different partners. Dynamic conformational switching among different structural sub-states favors the adaptation to the different shapes of the partners. Such conformational changes are induced by several biochemical factors, including ligand-binding. Understanding the mechanisms that underlie the regulation of functional dynamics by small-molecule ligands, at atomic levels of resolution, may provide new opportunities for the discovery of leads that target regulatory proteins.

In this review, we have reported our views and approaches to the development of novel and accurate physical-chemistry based models for the characterization of the salient aspects of the ligand-regulated dynamics of Hsp90, and on the exploitation of such new knowledge for the rational discovery of inhibitors of the chaperone. The discovered molecules showed interesting functional activities in different experimental tests, although they are not yet optimized to be considered as possible drug-candidates.

We suggest that it is now possible to include fully atomistic descriptions of protein dynamics in the drug-discovery process. In this context, the new fundamental challenge is identifying privileged structures capable of selectively interfering with key functional sub-states of proteins involved in signaling pathways. In a more general perspective this would allow the possibility to rationally design small molecules that tune, and not only inhibit, entire signaling cascades that control cell life.

To meet these challenges, it is fundamental to further develop and optimize new computational chemistry methods. The promise of our recent theoretical progresses in the identification of functional sub-states from the analysis of protein internal dynamics and energetics, combined to the growing increase in computer power and in the constant improvement of force fields for MD simulations, support the feasibility of strategies aimed at merging highly detailed computational analyses with experimental design methods to increase the chemical diversity space of active leads. This will allow to enhance our understanding of complex biomolecular systems and increase the efficiency with which appropriate chemical modulators of signaling pathways can be identified.
